# Mucosa plication reinforced colorectal anastomosis and trans-anal vacuum drainage: a pilot study with preliminary results

**DOI:** 10.1007/s13304-021-01105-4

**Published:** 2021-06-05

**Authors:** Alexander Ferko, Juraj Váňa, Marek Adámik, Adam Švec, Michal Žáček, Michal Demeter, Marián Grendár

**Affiliations:** 1 Department of Surgery and Transplant Centre, Jessenius Medical Faculty in Martin, Comenius University in Bratislava, University Hospital Martin, Martin, Slovak Republic; 2 Department of Surgery, Faculty Hospital Žilina, Žilina, Slovak Republic; 3 Department of Gastroenterology, Jessenius Medical Faculty in Martin, Comenius University in Bratislava, University Hospital Martin, Martin, Slovak Republic; 4 Laboratory of Bioinformatics and Biostatistics, Jessenius Medical Faculty in Martin, Biomedical Center Martin, Comenius University in Bratislava, Martin, Slovak Republic; 5 Františka Komárka 865/6, 503 11 Hradec Králové, Czech Republic

**Keywords:** Rectal cancer, Low anterior resection, Anastomotic dehiscence

## Abstract

Dehiscence of colorectal anastomosis is a serious complication that is associated with increased mortality, impaired functional and oncological outcomes. The hypothesis was that anastomosis reinforcement and vacuum trans-anal drainage could eliminate some risk factors, such as mechanically stapled anastomosis instability and local infection. Patients with rectal cancer within 10 cm of the anal verge and low anterior resection with double-stapled technique were included consecutively. A stapler anastomosis was supplemented by trans-anal reinforcement and vacuum drainage using a povidone-iodine-soaked sponge. Modified reinforcement using a circular mucosa plication was developed and used. Patients were followed up by postoperative endoscopy and outcomes were acute leak rate, morbidity, and diversion rate. The procedure was successfully completed in 52 from 54 patients during time period January 2019–October 2020. The mean age of patients was 61 years (lower–upper quartiles 54–69 years). There were 38/52 (73%) males and 14/52 (27%) females; the neoadjuvant radiotherapy was indicated in a group of patients in 24/52 (46%). The mean level of anastomosis was 3.8 cm (lower–upper quartiles 3.00–4.88 cm). The overall morbidity was 32.6% (17/52) and Clavien–Dindo complications ≥ 3 grade appeared in 3/52 (5.7%) patients. No loss of anastomosis was recorded and no patient died postoperatively. The symptomatic anastomotic leak was recorded in 2 (3.8%) patients and asymptomatic blind fistula was recorded in one patient 1/52 (1.9%). Diversion ileostomy was created in 1/52 patient (1.9%). Reinforcement of double-stapled anastomosis using a circular mucosa plication with combination of vacuum povidone-iodine-soaked sponge drainage led to a low acute leak and diversion rate. This pilot study requires further investigation.

**Registered at ClinicalTrials.gov.**: Trial registration number is NCT04735107, date of registration February 2, 2021, registered retrospectively.

## Introduction

Rectal resection for cancer is still associated with considerable morbidity. Acute leak (AL) is probably the most serious complication and is associated with increased postoperative mortality, with long-term consequences, such as a negative impact on function and oncological outcomes [1–3]. A proportion of patients with AL end up with an unplanned definitive stoma [4]; and increased economic costs associated with the treatment of complications and prolonged stays in the ICU should be taken in account [5, 6].

Published data on the occurrence of this complication are heterogeneous and greatly depend on the definition used, the design of the study [7–9] the duration of the study, and the composition of the group of patients studied [10, 11]. Very often, leaks that are asymptomatic [11] and leaks that are diagnosed after stoma closure are not accurately reported. The incidence of AL can reach 20–30% [2, 12]; and, if we only focus on the group of patients operated on for middle and lower rectal cancer, the leak rate may be even higher.

Many papers have been published analyzing preoperative, intraoperative, and postoperative factors associated with the development of AL [13–15]. Male gender, neoadjuvant radiotherapy, and low localization of anastomosis are generally accepted risk factors for AL development [16]. Surgeon experience is another factor which plays a very important role, and not much is written about it [17]. Surgeon experience is difficult to measure and adopt into risk calculation modeling; however, surgeons in high-volume centers present AL rates below 5% [18] and even these satisfactory results are associated with a high diversion rate [16, 19].

The scientific literature is yet to confirm a preference or differentiate between open, laparoscopic, robotic, or trans-anal approaches in the reduction of the incidence of this unpleasant complication [20–22]. Similarly, research in the field of leak prevention focuses in many directions [23]: stapled anastomosis replacement or modification [24, 25]; intraluminal biodegradable sheath application [26]; the colon microbiome influence [27–29]; and postoperative de-tension of the colon above an anastomosis [30, 31]. Various types of anastomosis reinforcement [32–37] have also been tested experimentally and clinically.

We investigated the intraoperative factors associated with AL that may be preventable. We initially needed to determine accurate leak rates in our patient group, including asymptomatic leaks. Therefore, the aims of our project were:

- 1. To determine the occurrence of an acute leak in patients operated on for rectal cancer up to 10 cm. Further, to specify the location and morphology of stapler line disruption in patients diagnosed with AL [38].
- 2. To verify the effect of modified trans-anal reinforcement in combination with trans-anastomotic vacuum drainage on the occurrence of acute leaks.

## Materials and methods

The study was approved by the ethics committee of our institution (Jessenius Medical Faculty in Martin, Slovak Republic) and was conducted in accordance with the Declaration of Helsinki.

### Inclusion criteria

**Fig. 1:**
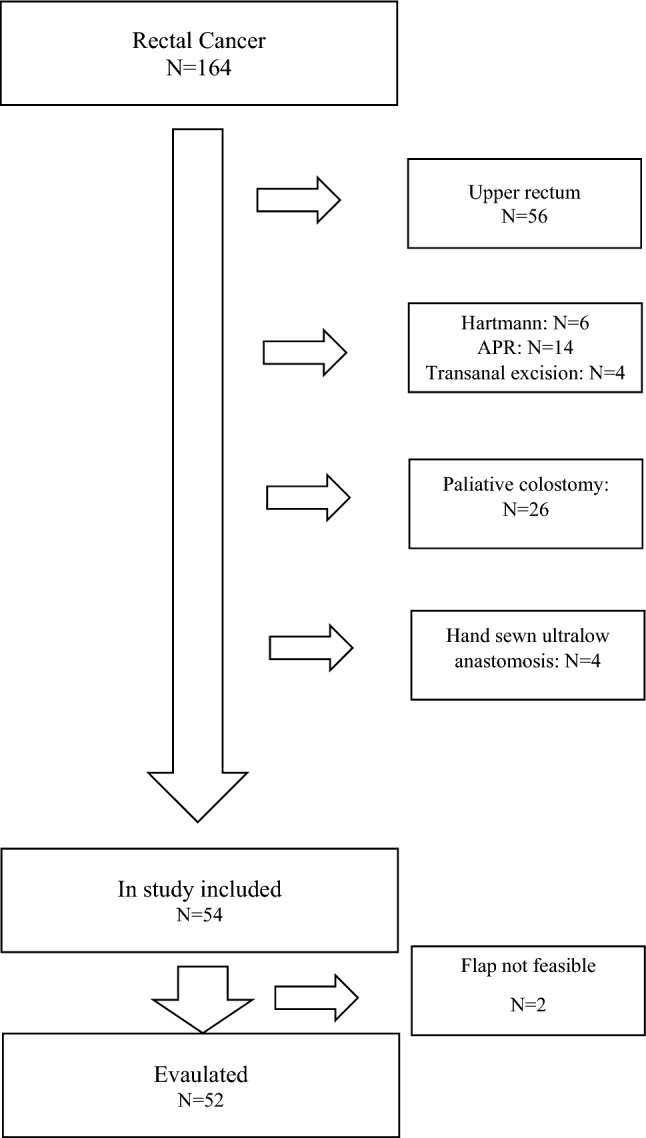
Patient flow chart

All patients provided written informed consent. The study included consecutive patients older than 18 years who had low anterior resection of the rectum and anastomosis performed by double-stapler technique, for rectal cancer located within 10 cm from the anal verge (Fig. 1).

All patients had undergone standard preoperative diagnostic evaluation, e.g., colonoscopy, endorectal sonography, or pelvic magnetic resonance imaging. Nutrition screening was performed in all patients. If patients had undergone neoadjuvant chemoradiotherapy (CHRT), restaging was performed within 6 weeks of CHRT completion, and surgery was performed 10 weeks after CHRT completion. For the surgical procedure, low anterior resection (LAR) was performed by experienced surgeons who perform more than 50 rectal procedures per year and have sufficient expertise in minimally invasive surgery. Oral bowel preparation was used preoperatively and antibiotics were administered according to protocol.

### Surgical technique

The procedure standard (descending colon blood perfusion, tension-free anastomosis, safely performed stapled anastomosis and reinforcement, and safely performed mucosal flap) was defined. Simultaneous checkpoints to control milestones were identified and methodology of their documentation (video, photography) was defined. The purpose was to achieve demonstrable control over the individual steps during the surgical procedure (Table 1).

**Table 1:** Surgical procedure standardization

Milestone	Checkpoint		Objectivisation
	Abdominal phase	Trans-anal phase	
Left colon blood perfusion	Checkpoint 1: pulsating marginal artery blood flow		Videorecord
Left colon blood perfusion	Checkpoint 2: transected colon mucosa is light red or pink color and fresh light red capillary bleeding is present		Videorecord
“Tension free” colorectal anastomosis	Checkpoint 3: colon lies free down in sacral excavation		Videorecord/photography
Left colon blood perfusion		Checkpoint 4: colon mucosa is light red or pink colored, contact fresh bleeding is present	
Stapled anastomosis integrity		Checkpoint 5: no defect in stapler line	Photography
“Tension free” colorectal anastomosis		Checkpoint 6: prolapsing “floppy colonic mucosa” is present	Photography
Circular mucosa flap was safely created		Checkpoint 7: tension-free flap with no tears and haematoma	Photography

Laparoscopic procedures were performed in the Lloyd-Davies position, using the 4-port technique. During the abdominal phase, dissection was guided by a medio-lateral approach. A high tie of the AMI was performed in all patients. Dissection was performed medio-laterally and down to the pelvic floor according to the principles of TME. The rectum was transected using an endostapler after lavage with Betadine solution (Egis Pharmaceuticals, PLS, Budapest, Hungary). Furthermore, the splenic flexure was fully mobilized using a combination of medio-lateral and lateral approaches. In most cases, the inferior mesenteric vein was divided.

The marginal artery was dissected and the character of arterial blood flow was carefully evaluated; pulsatile arterial blood flow was considered as sign of adequate colon perfusion (Checkpoint 1).

A specimen of tumor was pulled through the mini-laparotomy and resected. The descending colon was divided at the level of the distal part and the colonic mucosa was again evaluated with respect to blood perfusion; a light red or pink colored mucosa and fresh light red capillary bleeding were considered as signs of good colonic mucosa perfusion (Checkpoint 2). The colon needed to lie freely in the sacrum excavation and no tension was allowed on the mesenteric site. This was confirmed by lifting the colon ventrally from the sacrum at the promontory level after anastomosis construction (Checkpoint 3). The anastomosis was performed end-to-end using a double-stapler technique, strictly between the descending colon and rectum in a tension-free manner. A pelvic drain was left in place till the third postoperative day.

#### Trans-anal phase

**Fig. 2:**
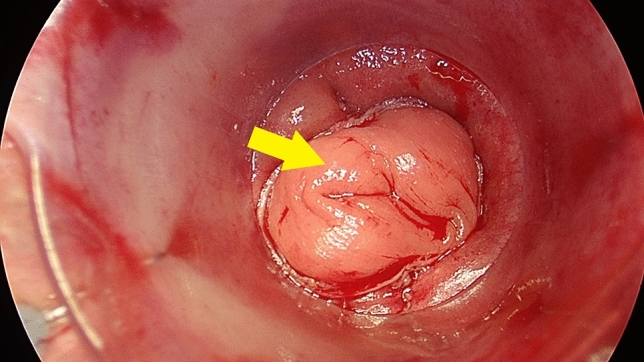
Trans-anal photography. Double-stapled anastomosis 15 mm from the upper edge of the anal ring. Colon mucosal prolapse (arrow) into the anastomosis is crucial for creation a tension-free mucosal flap

**Fig. 3:**
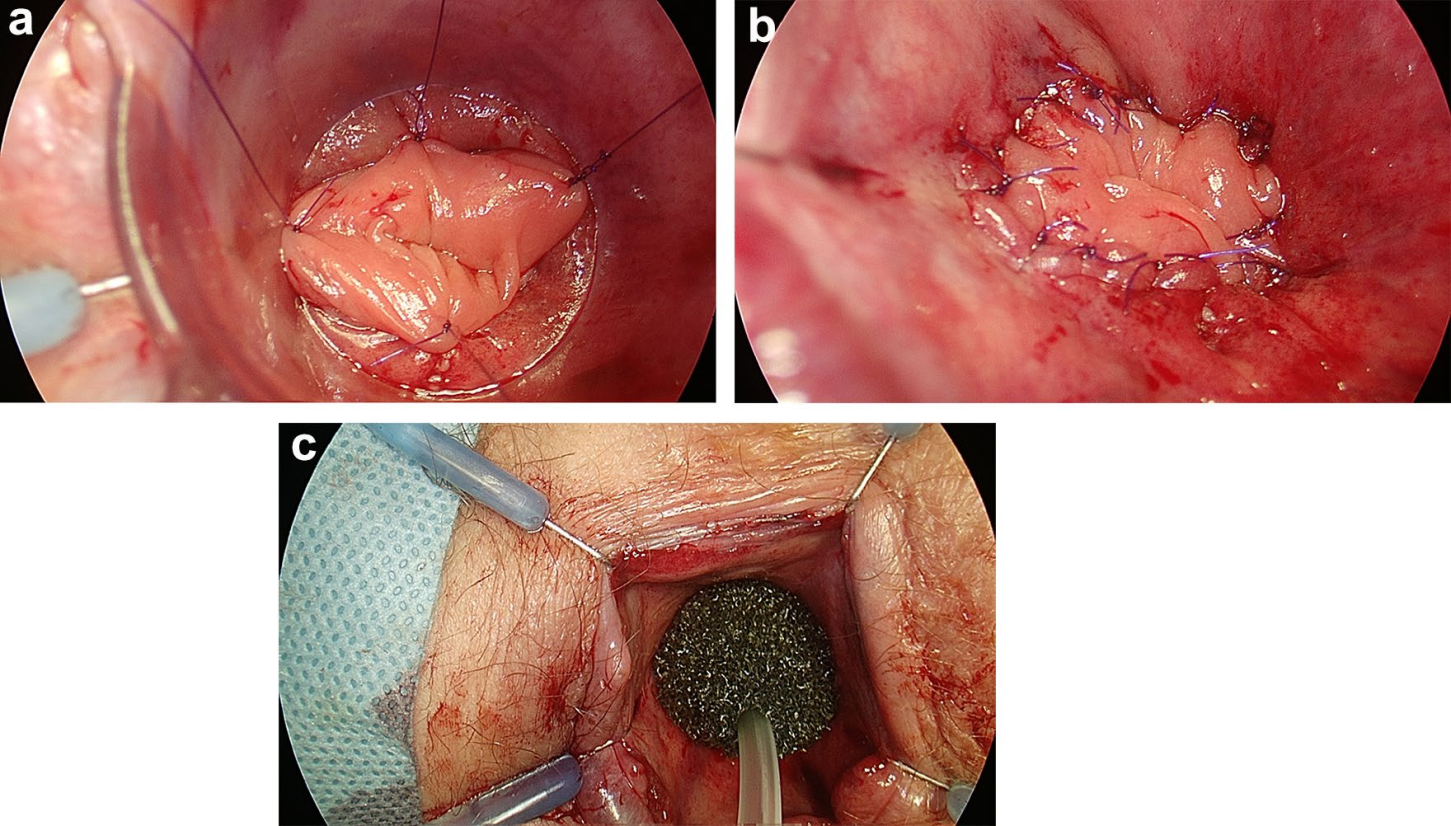
Trans-anal photography. Mucosa plication construction. Four stitches are first placed at 12, 3, 6, and 9 o’ clock (**a**); four stitches are subsequently applied to each quadrant (**b**); povidone-iodine-soaked sponge is introduced at the end of procedure (**c**)

As part of the trans-anal phase, a Lone Star retractor (Cooper Surgical, Inc. USA) and a plastic single use anoscope were applied. An initial, careful inspection and manual check of the stapler anastomosis integrity, the blood supply to the colonic mucosa, and signs of a tension-free anastomosis were performed (Fig. 2) (Checkpoint 4). The mucosal flap/plication was subsequently created using individual PDS II 5/0 sutures (polydiaxonone, Ethicon, Johnson & Johnson, USA): individual stitches were placed on each quadrant; and then another four stitches were applied in between (Fig. 3). It is important to note that the condition of the mucosal flap upon creation were signs of a floppy, prolapsing colonic wall into the anastomosis. Finally, a sponge soaked (Endo-SPONGE, B. Braun, Germany) with povidone-iodine (Betadine, Egis Pharmaceuticals, PLS, Budapest, Hungary) was introduced into the anastomosis (negative pressure 80–100 mm Hg). The trans-anal sponge drain was removed 24 h postoperatively.

#### Fecal diversion

The decision on diversion was based on intraoperative checkpoint adherence: when Checkpoint 6 and 7 were not fulfilled, an ileostomy was created.

### Follow-up

The data regarding the type of procedure, type of anastomosis, stapler diameter, the number of stapler cartridges used, dissection of the mesenteric blood vessels, and complete histopathology were collected prospectively. CRP levels were assessed on the third and fifth day after surgery.

Patients were followed up for 3 months, and postoperative endoscopy was performed before discharge, usually on postoperative day 7, 1 month after surgery, and 3 months after surgery.

### Statistics

The data were explored and analyzed in R [38], ver. 4.0.2. The number of patients at a level of a factor, together with the percentage of patients at the level of the factor was used to summarize the data.

## Results

Patients with mid- and low-rectal cancer were included in the study (Table 2). The mean age of patients was 61 years (lower–upper quartiles 54–69 years). There were 38/52 (73%) males and 14/52 (27%) females; the neoadjuvant radiotherapy was indicated in a group of patients in 24/52 (46%). The mean level of anastomosis was 3.83 cm (lower–upper quartiles 3.00–4.88 cm) from anal verge and 2.00 cm (lower–upper quartiles 1.00–2.00 cm) from upper edge of sphincters. All operations were performed minimally invasively (100%). The overall mean operative time was 255 min (lower–upper quartiles 225–277 min), the mean perineal phase time was 25 min (lower–upper quartiles 20–30 min).

**Table 2:** Patient group characteristic

	Intervention group* n* = 52
Age	61 (54.69)^1^
Sex	
Female	14 (27%)
Male	38 (73%)
ASA	
2	22 (42%)
3	30 (58%)
4	0
Smoking	8 (15%)
Cardiovascular comorbidity	27 (52%)
Corticosteroids	1 (2%)
Diabetes mellitus	5 (10%)
BMI	
pT	
pT0	5 (9.6%)
PTis	0 (0%)
PT1	4 (7.7%)
PT2	16 (31%)
pT3	24 (46%)
pT4a	2 (3.8%)
pT4b	1 (1.9%)
pN	
pN0	28 (54%)
pN1a	12 (23%)
pN1b	7 (13%)
pN1c	1 (1.9%)
pN2a	2 (3.8%)
pN2b	2 (3.8%)
pM	
pMX	0 (0%)
pM0	49 (94%)
pM1	3 (5.8%)
Tumor localization	
< 10 cm	46 (88%)
< 5 cm	6 (12%)
Neoadjuvant radiotherapy	
No	28 (54%)
Yes	24 (46%)
Diversion ileostomy	
No	51 (98%)
Yes	1 (1.9%)
Surgical approach	
Open	0 (0%)
Lap	52 (100%)
Robotic	0 (0%)
Anastomosis level	4.00 (3.00, 4.88)^1^
Stapler diameter	
< 27	0 (0%)
27–29	20 (38%)
29–33	32 (62%)
Vessel dissection	
A. rectalis supperior	0 (0%)
A. mesenterica inferior	52 (100%)
A. colica sinistra	0 (0%)
Morbidity	
No	35 (67%)
Yes	17 (33%)
Acute leak	
No	51 (98%)
Yes	1 (2%)
Dehiscence grade	
Grade A	0 (0%)
Grade B	0 (0%)
Grade C	1 (2%)
Dindo–Clavien	
I	4 (7%)
II	10 (19%)
III b	3 (6%)
Excision quality	
I	36 (72%)
II	11 (22%)
III	3 (6%)
Unknown	2 (4%)
pCRM	
> 1 mm	46 (90%)
< 1 mm	5 (9.8%)
Unknown	1 (2%)
Resection margins	
R0	51 (98%)
R1	1 (2%)
Unknown	0 (0%)

^1^Statistics presented: *n* (%); median (IQR)

### Morbidity and mortality

No patients died postoperatively and no intraoperative complication was recorded. Endoscopic examination before discharge home was completed in all 52 patients (100%). Patients underwent control endoscopy 1 month (50/52 patients) and 3 months (49/52 patients) after surgery. The overall morbidity was 33% (17/52) and serious complications, Clavien–Dindo ≥ 3 grade, appeared in 3/52 (5.7%) patients (Table 3). No loss of anastomosis was recorded, 3 (5.7%) anastomotic complications (2 symptomatic, 1 asymptomatic) were recorded, at all. The symptomatic anastomotic leak was recorded in 2/52 (3.8%) patients, one acute leak a one recto-vaginal fistula. A recto-vaginal fistula was diagnosed 1 month after surgery in one female. A retrospective analysis of the recorded video revealed a technical error of the surgeon in performing the stapler anastomosis. Another patient was diagnosed with asymptomatic blind chronic anastomotic fistula 3 months after surgery, which did not require any treatment intervention.

**Table 3:** Postoperative morbidity

Dindo–Clavien classification	Patients *n* = 52
Grade I	
Superficial wound infection	1
Postoperative diarrhea required rehydration	2
Superficial vein thrombophlebitis	1
Grade II	
Urinary retention requires catheter placement	3
Deep vein thrombosis	1
Postoperative anemia required transfusion	2
Postoperative GIT paralysis lasting > 5 days	1
Urosepsis	1
Colitis (*Clostridium difficile*)	1
Postoperative fever required ATB (leek not proven)	1
Grade III b	
Acute leak, grade C	1
Recto-vaginal fistula	1
Postoperative ileus	1
All	17/52 (33%)

Diversion ileostomy was placed on 1/52 (1.9%) patient. This ileostomy was performed due to uncertainty about the quality of the reinforcement performed (Checkpoints 6 and 7).

### Protocol violation and patient exclusion

A low anterior resection with double-stapled anastomosis for extraperitoneal rectal cancer was indicated in 54 of 186 patients (Fig. 1). In two patients, no reinforcement was performed due to anal canal stenosis after hemorrhoid surgery, and in another patient due to obesity (BMI 42). One of these patients developed AL, which was treated by ileostomy and trans-anal vacuum drainage. The standardized protocol was violated in one patient; the colon was not adequately cleaned before the surgery. Immediately after the operation a massive defecation of stool with a temporary obstruction of the colon above the sponge occurred. This patient was excluded from the study. Acute B grade leak was observed and treated with trans-anal vacuum drainage in the postoperative period. The patient did not lose the anastomosis.

## Discussion

We showed that reinforcement of a double-stapled anastomosis using mucosal flap with the combination of vacuum povidone-iodine-soaked sponge drainage led to a significant decrease in AL and diversion rate. However, AL is a complication that still deserves considerable attention.

The search for optimal treatments should focus on rapid pelveoperitonitis or peritonitis treatment and the rescue of sphincters, if possible. No less important is the search for factors associated with the leak formation. Analysis of the photographic documentation of endoscopic findings on the anastomosis from our previous study [38] allowed us to observe signs of local stress on the anastomosis, ischemic changes, loose stapler clamps, as well as other pathological findings, such as fibrosis and inflammatory polyps. These signs suggest a healing disorder and “restlessness” in the area of the stapler anastomosis, which is associated with local ischemia or local infection. The result is a defect in the anastomosis with the spread of infection to the perianastomotic space or an exacerbated and scarring reaction. These findings led us to our hypothesis formulation. Five basic pathogenic moments were identified:

- 1. The blood supply to the large intestine, and especially the section above the anastomosis, is very important and must be verified intraoperatively.
- 2. Tension-free colorectal anastomosis is an indispensable condition for successful completion of the procedure.
- 3. Double-stapler anastomosis poses a higher risk of mechanical disruption of the stapler line; therefore, trans-anal reinforcement of the anastomosis may play an important role in its prevention.
- 4. A colorectal anastomosis is a contaminated wound and is at risk of bacterial invasion during the first 24 h, like any other contaminated wound.
- 5. Endo-anal trans-anastomotic drainage may play a role in reducing the risk of leakage and de-tension of the colon above the anastomosis.

If we pause at the first two points of our hypothesis, there will probably be general agreement that blood flow to the colon and tension-free anastomosis are very important; however, it should be emphasized that tension-free anastomosis requires full mobilization of the splenic flexure, in contrast to resection of upper rectal tumors. This is consistent with Rink et al., who published the Delphi Consensus from the German expert meeting in 2020 [16]. However, full mobilization of the splenic flexure is technically demanding, sometimes significantly prolonging the operation, and is associated with risk of injury to the spleen, pancreas, or marginal collateral vessel. Surgeon training seems to be very important in this instance.

Mobilization of the splenic flexure requires division of the main vessels, AMI, and often the inferior mesenteric vein (VMI). This leads to a decrease in arterial perfusion [39], but also to poor left colon venous drainage. Another alternative to high AMI is division of the AMI distal to the origin of the left colic artery (LCA) (the so-called low AMI tie), which would ensure sufficient blood supply to the sigmoid colon. In this context, Guo et al. directly measured the pressure in the LCA and found that the mean arterial pressure in the ACS at low AMI ligation is higher than at high AMI ligation [40]. LCA dissection may be an alternative, especially in at-risk patients with sclerotic arterial disease. The question is whether dissection of the apical nodes is necessary and will be comparatively radical. At present, this method is not accepted as a standard and is the subject of further evaluation. Consideration of magistral vessel ligature is very important and knowledge of the anatomical variability of the vascular supply to the colon is necessary. The medial colic artery (MCA), which is the source of blood flow for ACS, may be completely absent in a large proportion of patients [41]. The medial collateral artery (Arc of Riolan), which accompanies VMI, occurs in about 7% of patients, and might significantly complicate full splenic flexure mobilization. Its interruption might lead to severe ischemia of the left colon. Intraoperative identification of vascular variability is difficult and often impossible in obese patients.

Therefore, to gain control over the blood supply to the large intestine, checkpoints have been set in our standard (Table 2). We relied on old surgical principles: control of pulsatile flow on the marginal artery and control of the mucosa and its blood supply. Although we have infrared camera technology, we have not used it as the standard in our study. The interpretation probably requires a quantitative approach [42], especially in patient with VMI division and impaired venous blood drainage of the left colon.

As regards the next point of our hypothesis, the double-stapling technique of colorectal anastomosis, previously published work on the topic of reinforcement and modifications of the stapler anastomosis [25, 43] may indirectly indicate doubts about the safety of this technique [44, 45]. Reinforcement of a stapled or double-stapled anastomosis of various technical designs is appearing with increasing frequency in the literature and remains the subject of ongoing studies. Individual authors have used various techniques, such as glues [33, 36], bio absorbable pads [34], intracorporeal applied sutures [32], or trans-analy applied sutures [35, 37, 46]. We started with trans-anal suture reinforcement within our hypothesis as we believed that the reinforcement suture would relieve local tension between the anastomosed colon and the rectum. We subsequently discovered that the transmurally applied sutures technique is difficult and can lead to injuries to the wall of the colon and rectum around the anastomosis. Therefore, we modified this technique. The mucosal flap was created with the assumption that well-perfused tissue, rich with immunocompetent cells, covers entry to the wound and decrease risk of bacterial deep invasion. This might lead to local inter-staple infection reduction in the first 24 h after surgery. Importantly, however, is that the mucosal flap should be performed with respect to the tension-free technique.

The last pillar of our hypothesis is vacuum drainage using a sponge infused with an antiseptic solution. This part of the hypothesis is based on several findings. A colorectal anastomosis is classified as a contaminated wound. The first (exudative) phase of healing is characterized by the lack of immunocompetent cells, neutrophils, in the wound which provide immune protection against bacterial invasion. The wound is impermeable to bacteria after 24 h [47]. Therefore, the reduction of bacterial load at the time of wound formation may be important in the development of local infection between the stapler clamps [27].

Another pathophysiological consideration is the intraluminal pressure in the large intestine above the anastomosis. Intestinal decompression above the anastomosis may reduce the risk of AL [30, 31]. Endosponge is used for intraluminal treatment of acute leakage [48], but its preventive use in this indication is no longer fully known. Therefore, we must rely on data from studies where vacuum therapy has been tested in the prevention of early infection [49–51]. However, the conclusions are ambiguous and it cannot be argued that vacuum therapy should be routinely used to prevent early infection in colorectal surgery [52]. Similarly, we cannot unequivocally say that the applied vacuum leads to a reduction in the bacterial load in the wound area [53]. What is interesting, however, is that vacuum therapy can promote neovascularization, and thus healing. Labler et al., point to higher local concentrations of IL-8 and VEGF, and thus higher leukocyte attraction and improved promotion of neovascularization [51]. Any trans-anal drainage can only be applied to patients with a perfectly prepared and clean colon; otherwise, the unpleasant complication of obstruction may result, as seen in our one patient.

The indication of fecal diversion in rectal resection may not only have medical reasons. Many articles regarding this have been published [19], and to state that ileostomy can alleviate the clinical severity of AL but probably does not prevent it. In our cohort, we indicated ileostomy in one patient out of 52 (1.9%). The reason was the uncertainty about mucosal plication vitality after the completion of reinforcement (hematoma and mucosa crack) (Checkpoints 6 and 7).

Our study had limitations in terms of the number of patients and functional outcomes monitoring. Although the trans-anal phase of the operation did not last longer than 20 min on average, this requires further evaluation.

Our pilot results would imply that standardization of surgery, perioperative control of colonic blood flow, tension-free anastomosis, trans-anal control of anastomosis integrity, and reinforcement with vacuum drainage led to a low acute leak and low diversion rate. Our results from this preliminary study are promising and require further investigation. At present, we are unable to clearly identify the value and weight of each individual step, and believe that this is a combined effect of all individual measures. What needs to be emphasized, however, is that any trans-anal intervention cannot replace proper surgical technique. Surgeon experience is an irreplaceable factor.

## Data Availability

FERKO, Alexander (2021), “Mucosal Flap Reinforced Colorectal Anastomosis and Trans-Anal Vacuum Drainage”, Mendeley Data, V1. http://dx.doi.org/10.17632/7x93t5hv6p.1.

## Code availability

Declared in section Material and methods, page 5 in Manuscript file.
